# Bond Strength and Deflection of Four Types of Bonded Lingual Retainers

**DOI:** 10.1155/2022/1707520

**Published:** 2022-02-24

**Authors:** Amin Golshah, Shirin Asadian Feyli

**Affiliations:** ^1^Department of Orthodontics, School of Dentistry, Kermanshah University of Medical Sciences, P. O. Code 6715847141, Kermanshah, Iran; ^2^Students Research Committee, School of Dentistry, Kermanshah University of Medical Sciences, P. O. Code 6715847141, Kermanshah, Iran

## Abstract

**Objectives:**

This study aimed to assess the bond strength and deflection of four types of bonded lingual retainers.

**Materials and Methods:**

In this in vitro, experimental study, 160 extracted, mandibular incisors were mounted in acrylic blocks in sets of two and randomized into four groups for bonding of 1.0.010 × 0.026-inch Bond-A-Braid®, 0.012 × 0.027-inch Retanium®^TM^, 0.038 × 0.016-inch Ortho FlexTech®, and 0.0175-inch three-strand retainer wires; 15 mm of passive wire was adhered to the lingual tooth surface using Transbond XT composite. The shear (SBS) and tensile (TBS) bond strength values were measured. The adhesive remnant index (ARI) score and deflection of wires were also determined under a stereomicroscope. Data were analyzed by the chi-square test and ANOVA.

**Results:**

The four groups were significantly different regarding the ARI scores (*P* < 0.05). Significant differences were noted between the three-strand and all other groups in deflection (*P* < 0.05). The Retanium group had significant differences with other groups in peak SBS (*P* < 0.05). A significant difference was found between the Retanium and Ortho Flex groups in break SBS (*P* < 0.05). Significant differences were also reported between the three-strand and all other groups in peak TBS (*P* < 0.05).

**Conclusion:**

The Retanium retainer had the maximum SBS, while the three-strand retainer had the maximum TBS. The three-strand and Retanium wires can probably better tolerate intraoral forces and have higher resistance to fracture due to having higher TBS. Also, the three-strand wire had lower deflection rate, which highlights its higher resistance to occlusal forces. Retanium and Ortho FlexTech wires had the most favorable failure modes.

## 1. Introduction

Maintaining the alignment of the teeth after orthodontic treatment is highly important [1]. The length of dental arch decreases, and consequently, the crowding of anterior teeth increases with aging [2, 3]. Thus, the use of permanent retainers appears to be the only way to maintain the ideal alignment of the teeth after orthodontic treatment [2, 3]. Several factors are responsible for unwanted tooth movement after orthodontic treatment such as regeneration of periodontal tissue [4], changes related to growth and development after treatment [5], and type of treatment performed [6]. To prevent unwanted tooth movements, retainer wires are connected to the lingual surface of the maxillary and particularly mandibular incisors [1]. Many factors can compromise the optimal function of the retainers adhered to the teeth such as debonding at the enamel-composite interface (adhesive failure), debonding at the wire-composite interface (cohesive failure), a combination of both (mixed failure), and tension fracture of the wire [1, 7, 8]. Cohesive failure is among the most common types of failure [1]. Since bonded lingual retainers should remain in the oral cavity for long periods of time, their success rate must be maximized. Wire selection can play a fundamental role in this regard [9]. Moreover, lingual retainers should be flexible and have high bond strength and optimal resistance against unwanted deflection [10, 11].

Many types of bonded lingual retainers are commercially available, made of stainless steel or titanium. Nonetheless, information regarding their properties is limited, making it difficult to select an ideal retainer to achieve the treatment goals.

This study was carried out aiming to assess the debonding force of four types of bonded lingual retainers, namely, 0.0175-inch three-strand retainer, Bond-A-Braid, Ortho FlexTech, and Retanium under shear and tensile forces.

## 2. Materials and Methods

A total of 160 human mandibular incisors, extracted due to hopeless periodontal prognosis, were collected for this in vitro, experimental study. The sample size was calculated to be 20 in each group (a total of 80 in all four groups) considering *α* = 0.05, 1-beta = 90%, standard deviation of shear bond strength (SBS) of Bond-A-Braid and PentaOne to be 19.43 and 8.15N, respectively, and *d* (accuracy) = 16. Thus, 80 acrylic blocks (each containing two incisor teeth) were evaluated in this study [9].

The collected teeth had no caries, cracks, or anomaly. The tissue residues were removed by a scaler, and the teeth were disinfected by immersion in 1% thymol solution [9, 12]. To fabricate our experimental model, each pair of incisor teeth was mounted in one acrylic block such that their interdental contact and position simulated their position in the dental arch. The teeth were mounted in acrylic resin, such that their longitudinal axis was perpendicular to the surface of the acrylic block. Autopolymerizing acrylic resin was poured around the roots to the level of their cementoenamel junction. To simulate the periodontal ligament, each root was wrapped in a thin layer of silicone. It should be noted that all blocks had the same dimensions [1]. Acrylic blocks were then randomized into four groups (*n* = 20) as follows:

  Group 1: 0.010 × 0.026-inch wire (Bond-A-Braid®, Reliance Orthodontic Products, Itasca, IL, USA)
  Group 2: 0.012 × 0.027-inch wire (Retanium™®, Reliance Orthodontic Products, Itasca, IL, USA)
  Group 3: 0.038 × 0.016-inch wire (Ortho FlexTech®, Reliance Orthodontic Products, Itasca, IL 60143, USA)
  Group 4: 0.0175-inch three-strand wire (Ortho Technology, Tampa, Florida, USA)

The lingual-surface enamel was polished with fluoride-free pumice paste, and after rinsing and drying, it was etched with 37% phosphoric acid gel (Transbond XT etching gel system; 3M Unitek, Monrovia, CA, USA) for 30 seconds [9, 13]. After rinsing and drying of the tooth surface, the bonding agent (Transbond XT system; 3M Unitek, Monrovia, CA, USA) was applied [9] and cured for 20 seconds [14]. Next, 15 mm of passive wire was adhered to the tooth surface using light-cure composite resin (Transbond XT adhesive; 3M Unitek, Monrovia, CA, USA) [1]. The composite resin was light-cured for 20 seconds [15]. Prior to curing, the midpoint of the wire was marked and positioned at the interdental contact area. The wire was paralleled to the surface of the acrylic base. Also, the amount of composite for use was standardized (equalized) by using a dome-shaped instrument (Mini-Mold™; Ortho-Care Ltd., Bradford, West Yorkshire, UK) [9]. After bonding of the retainers to the teeth, the assemblies were immersed in distilled water at 37°C for 24 hours [1]. Subsequently, their SBS was measured by a universal testing machine (Instron Co., Canton, MA, USA). To apply shear force, a custom-made chisel was used. The chisel blade was adjusted such that it had no contact with the teeth at the time of load application. Vertical load was applied to the previously marked midpoint of the wire by the chisel blade [1, 9]. The crosshead speed of the device was adjusted at 2 mm/minute, and the load causing debonding of the retainer was recorded [16]. The adhesive remnant index (ARI) scores were determined by quantifying the adhesive remnants on the enamel surface where the debonding occurred under a stereomicroscope (Leica 245E; Buffalo Grove, IL, USA) at x20 magnification [1, 17]. The classification system suggested by Artun and Bergland [17] (scores 0–3) was used for this purpose as follows:

  Score 0: no adhesive remnant on the enamel surface
  Score 1: <50% of adhesive remaining on the enamel surface
  Score 2: >50% of adhesive remaining on the enamel surface
  Score 3: all adhesive remaining on the enamel surface [1, 13, 18].

Moreover, the wire deflection after debonding was assessed under the stereomicroscope at x20 magnification [1, 19]. To measure the tensile bond strength (TBS), 40 acrylic blocks with the same size as the blocks used for the SBS test were prepared, and a hole measuring 2 × 3 mm was created at the center of each block. Next, 10 wires with 10 cm length were separated from each of the four retainer types and adhered to the center of acrylic block using composite resin. These blocks were then subjected to tensile force at a crosshead speed of 10 mm/minute in the universal testing machine, and the results were compared [9].

All statistical analyses were performed by SPSS version 26. Normal distribution of the data was evaluated by the Shapiro–Wilk test. ANOVA was applied to analyze the normally distributed data, while the chi-square test was used to analyze the data with nonnormal distribution and qualitative variables. Level of significance was set at 0.05.

## 3. Results

### 3.1. ARI Scores

**Figure 1:**
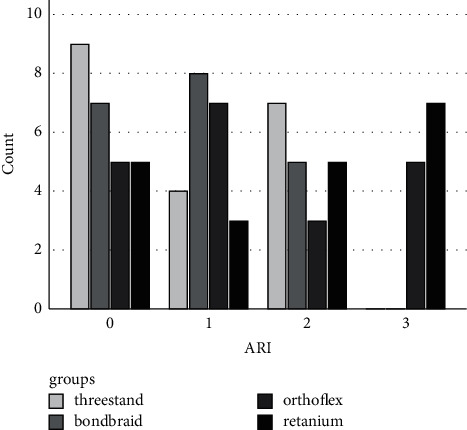
Distribution of ARI frequency among study groups.

Table 1 and Figure 1 show the frequency of ARI scores in the four groups. The chi-square test was used to compare the ARI scores among the four retainer groups, which revealed a significant difference (*P* < 0.05). The ARI score 3 had the highest frequency in the Retanium and Ortho Flex groups (58.3% and 41.7%, respectively). The ARI score 1 had the highest frequency in the Bond-A-Braid group (36.4%), while the ARI score 2 had the highest frequency in the three-strand group (35.0%).

### 3.2. Deflection

**Figure 2:**
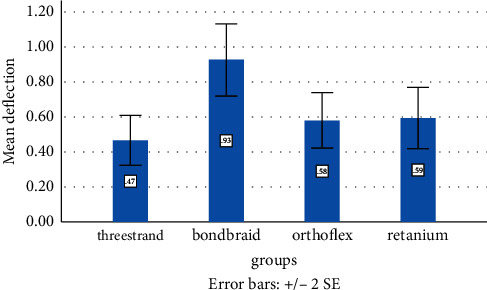
Mean deflection rate in the four groups.

According to the Shapiro–Wilk test, all groups had normal distribution of deflection data except for one group. The Box-Cox conversion feature of the Minitab software was applied to stabilize the variances. After the conversion, the normality test was repeated, and all groups were found to have normal data distribution. ANOVA was used to analyze deflection, which revealed a significant difference in this respect among the four groups. Pairwise comparisons by Tukey's test were then applied, which showed significant differences between the three-strand group and all other groups (*P* < 0.05, Table 2, Figure 2).

It should be mentioned that 5 specimens in the Ortho Flex group and 7 specimens in the Retanium group broke.

### 3.3. SBS Test

**Figure 3:**
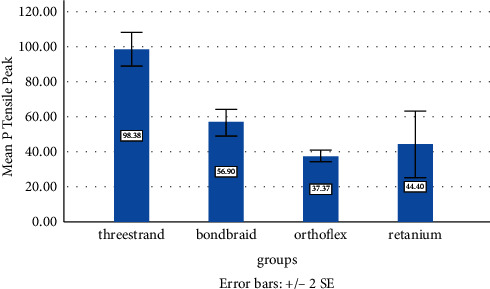
Mean shear bond strength at peak in the four groups.

According to the Kolmogorov–Smirnov test, the SBS data had a normal distribution in all four groups. Thus, ANOVA (parametric test) was applied to compare the SBS of the four groups. A significant difference was found among the four groups in the peak SBS values (*P* < 0.05). Thus, pairwise comparisons were performed by Tukey's test, which revealed significant differences between the Retanium and other groups (*P* < 0.05, Table 3, Figure 3).

ANOVA also revealed a significant difference in the break values among the four groups (*P* < 0.05), and Tukey's test showed a significant difference between the Retanium and Ortho Flex groups (*P* < 0.05, Table 4).

### 3.4. TBS Test

**Figure 4:**
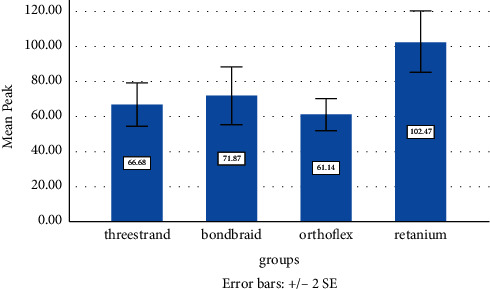
Mean tensile bond strength at peak in the four groups.

The Kolmogorov–Smirnov test revealed normal distribution of the peak and nonnormal distribution of the break values in all four groups. However, since the assumption of homogeneity of variances was met and the groups had equal sample size, ANOVA was applied for the comparison of the four groups, which revealed a significant difference in the peak values among the four groups (Figure 4) and Tukey's test showed a significant difference between the three-strand group and all other groups (P<0.05)

To ensure accuracy of the test, analysis was repeated by a nonparametric test as well, which yielded the same result. Pairwise comparisons by Tukey's test revealed significant differences between the three-strand group and all other groups (*P* < 0.05, Table 5). The four groups were not significantly different regarding the break values (*P* > 0.05).

## 4. Discussion

### 4.1. Bond Strength

The present results indicated that the Retanium retainer had the maximum SBS while the three-strand retainer had the maximum TBS. In line with our findings, Samson et al. [10] demonstrated that the bond strength of the three-strand retainer was higher than that of Bond-A-Braid. In the present study, the three-strand retainer had a TBS of 98.38 N, which was slightly different from the value reported by Samson et al. [10], i.e., 107.17 MPa. This small difference can be due to different methodologies and use of different adhesives and composite resins. The SBS of Bond-A-Braid was found to be 56 N in both studies, which was significantly different from SBS of the three-strand retainer. Also, SBS of Bond-A-Braid in the present study was close to the values reported by Baysal et al. [9] and Radlanski and Zain [12] (64.3 MPa). The low SBS of Bond-A-Braid can be due to its flattened structure, while the three-strand retainer is made of braided circular cross-sectional wires. The latter wires have been more comprehensively studied than flattened wires, and despite having higher flexibility, they have higher strength and lower deflection [1].

In general, it is believed that orthodontic wires should have a TBS value >5–8 MPa (minimum force applied during mastication or other intraoral forces) [20]. Bonded orthodontic biomaterials should provide sufficient adhesion to withstand masticatory forces (minimum bond strength of 5–10 MPa); however, the bond strength should not be too high to avoid substrate loss following debonding (40–50 MPa). Therefore, ideal orthodontic biomaterials must have bonding forces in the range of 5–50 MPa, even if these values are mainly theoretical [21]. Nonetheless, lingual retainers are less subjected to intraoral forces; therefore, lower bond strength values may also be acceptable for them [20]. On the other hand, Cooke and Sherriff [1] believed that this value cannot be generalized to retainer wires since vertical forces applied to the retainer wires are not uniformly distributed along the wire length, resulting in generation of a combination of shear, tensile, and shrinkage forces along the wire, all at the same time [1]. It should be noted that some other parameters such as the technique of bonding and type of adhesive also play a role in fracture of retainers in the oral cavity [22]. However, addressing all these parameters was out of the scope of the present study. Thus, further studies on different types of retainer wires and different adhesives are required to confirm the present results.

### 4.2. Deflection

The current results indicated that the deflection of the three-strand retainer was lower than that of others. Two types of multistrand wires were evaluated in the present study, namely, Bond-A-Braid and three-strand, which are both made of stainless steel, and are composed of 8 and 3 braided strands, respectively. Two single-strand wires, namely, Retanium and Ortho FlexTech were also evaluated in this study; the first one is made of titanium (without nickel) and the second one is made of stainless steel. Bearn [23] suggested that the retainer wires should preferably have low thickness and multiple braided strands. Nagani [24] reported lower failure rate of multistrand retainers compared with reinforced retainers. Also, multistrand retainers have less adverse effects on the periodontal tissue (such as inflammation or gingival recession) and lower risk of caries [25]. Baysal et al. [9] demonstrated that deflection of Bond-A-Braid wire was higher than that of five-strand braided wire. Samson et al. [10] indicated that the three-strand wire experienced the lowest deflection upon application of a certain amount of load compared with Bond-A-Braid. In the present study, deflection of Bond-A-Braid (with 8 strands) was higher than that of three-strand wire, which can be attributed to the flattened nature of Bond-A-Braid wires.

### 4.3. ARI Score

In the present study, Bond-A-Braid had the maximum frequency of ARI score 1, which indicates that at the time of debonding, less than 50% of adhesive remained on the enamel surface in most cases. Regarding the three-strand wire, the ARI score 2 had the highest frequency (>50% of adhesive remaining on the enamel surface). The ARI score 3 (all adhesive remaining on the enamel surface) had the highest frequency in the Retanium and Ortho FlexTech groups. In fact, the bracket-adhesive interface is considered as the most favorable point of debonding such that maximum adhesive remains on the enamel surface. Thus, ARI scores 2 and 3 are the most favorable modes of failure in debonding of brackets and retainers because they minimize the risk of enamel fracture [26, 27].

Extracted human mandibular incisors were used in this study. Although the results would be closer to the clinical setting in case of using the human teeth, such teeth cannot be perfectly standardized in terms of lingual shape, size, degree of mineralization, and dental age. Since 20 teeth were allocated to each group in the present study, the effect of such confounding factors on the results was minimized [12, 28]. In this study, the roots were wrapped in silicone to simulate the periodontal ligament and its cushioning effect at the time of load application. By doing so, load application conditions were standardized for all teeth [1, 29]. Moreover, the same type of composite resin in the same amount and one type of adhesive with similar application steps were used in all groups for the purpose of standardization and elimination of confounding factors. All procedures in all groups were performed by the same operator to minimize errors. Also, the collected teeth were stored in distilled water at 37°C to simulate intraoral conditions.

In vitro studies have certain limitations since they cannot completely simulate the clinical setting; thus, generalization of their results to the clinical environment should be done with caution. Presence of saliva, humidity, and temperature alterations of the oral environment, frequent masticatory forces, pressure of the tongue, normal physiological movements of the teeth, and even the oral bacteria cannot be well simulated in vitro. On the other hand, properties of the periodontal tissue such as its viscoelasticity, periodontal ligament width, and alveolar bone cannot be simulated in vitro; thus, their effects on the properties of retainers cannot be investigated [10]. Clinical trials are required to find the most ideal retainer for use in the clinical setting. Also, further studies should focus on different types of retainer wires, adhesives, and bonding techniques that are required to further elucidate this topic.

## 5. Conclusion

The current results indicated different mechanical properties of commercially available retainer wires. The three-strand and Retanium wires can probably better tolerate intraoral forces and have higher resistance to fracture due to having higher TBS. Also, the three-strand wire had lower deflection rate than other wires, which highlights its higher resistance to occlusal forces. Thus, while being flexible, it prevents unwanted tooth movements. Moreover, in debonding of the Retanium and Ortho FlexTech wires, the entire adhesive, and in debonding of the three-strand wire, over 50% of adhesive remained on the enamel surface, indicating that enamel surface is less damaged during debonding.

## Figures and Tables

**Table 1:** Frequency of ARI scores in the four groups (*n* = 20).

Groups	Score 0	Score 1	Score 2	Score 3
Three-strand	9	4	7	0
Bond-A-Braid	7	8	5	0
Ortho Flex	5	7	3	5
Retanium	5	3	5	7

**Table 2:** Pairwise comparisons of the groups regarding deflection by Tukey's test.

Group (I)	Group (J)	Mean difference (I-J)	Std. error	*P* value	95% confidence interval	
					Lower bound	Upper bound
Three-strand	Bond-A-Braid	−1.94^*∗*^	0.17	≤0.001	−2.42	−1.46
	Ortho Flex	−1.60	0.18	≤0.001	−2.12	−1.08
	Retanium	−1.61^*∗*^	0.19	≤0.001	−2.15	−1.07

Bond-A-Braid	Ortho Flex	0.35	0.18	0.31	−0.17	0.86
	Retanium	0.33	0.19	0.38	−0.20	0.87

Ortho Flex	Retanium	−0.01	0.20	1.00	−0.58	0.56

^*∗*^The mean difference is significant at 0.05 level.

**Table 3:** Pairwise comparisons of the peak SBS values of the four groups.

Group (I)	Group (J)	Mean difference (I-J)	Std. error	*P* value	95% confidence interval	
					Lower bound	Upper bound
Three-strand	Bond-A-Braid	−5.19	10.06	0.95	−31.61	21.23
	Ortho Flex	5.54	10.06	0.95	−20.88	31.96
	Retanium	−35.79^*∗*^	10.06	≤0.001	−62.21	−9.37

Bond-A-Braid	Ortho Flex	10.74	10.06	0.71	−15.68	37.15
	Retanium	−30.59	10.06	0.02	−57.01	−4.17

Ortho Flex	Retanium	−41.33^*∗*^	10.06	≤0.001	−67.75	−14.91

^*∗*^The mean difference is significant at 0.05 level.

**Table 4:** Pairwise comparisons of the break SBS values of the four groups.

Group (I)	Group (J)	Mean difference (I-J)	Std. error	*P* value	95% confidence interval	
					Lower bound	Upper bound
Three-strand	Bond-A-Braid	0.99	9.23	1.00	−23.26	25.25
	Ortho Flex	13.47	9.23	0.47	−10.78	37.73
	Retanium	−15.06	9.23	0.34	−39.85	8.65

Bond-A-Braid	Ortho Flex	12.48	9.23	0.53	−11.77	36.73
	Retanium	−16.59	9.23	0.28	−40.85	7.66

Ortho Flex	Retanium	−29.07	9.23	0.01	−53.33	−4.82

^*∗*^The mean difference is significant at the 0.05 level.

**Table 5:** Pairwise comparisons of the peak TBS values of the four groups.

Group (I)	Group (J)	Mean difference (I-J)	Std. error	*P* value	95% confidence interval	
					Lower bound	Upper bound
Three-strand	Bond-A-Braid	41.48^*∗*^	8.02	≤0.001	19.89	63.07
	Ortho Flex	61.01^*∗*^	8.02	≤0.001	39.42	82.60
	Retanium	53.98^*∗*^	8.02	≤0.001	32.39	75.57

Bond-A-Braid	Ortho Flex	19.53	8.02	0.09	−2.06	41.12
	Retanium	12.50	8.02	0.42	−9.09	34.09

Ortho Flex	Retanium	−7.03	8.02	0.82	28.62	14.56

^*∗*^The mean difference is significant at the 0.05 level.

## Data Availability

The data used to support the findings of this study are available from the corresponding author upon request.
